# Draft genomes of *Citrobacter freundii* isolated from a Stillwater, Oklahoma wastewater plant

**DOI:** 10.1128/mra.00935-25

**Published:** 2025-11-05

**Authors:** Samiratu Mahazu, Lou Vanhauwaert, Sushim Gupta, Fares Z. Najar, Chelsea L. Murphy, Shiping Deng, Mark Krzmarzick, John E. Gustafson

**Affiliations:** 1 Department of Biochemistry and Molecular Biology, Oklahoma State University 7618, Stillwater, Oklahoma, USA; 2 High-Performance Computing Center, Oklahoma State University 7618, Stillwater, Oklahoma, USA; 3 Department of Plant and Soil Science, Oklahoma State University 7618, Stillwater, Oklahoma, USA; 4 Department of Civil and Environmental Engineering, Oklahoma State University 7618, Stillwater, Oklahoma, USA; Rochester Institute of Technology, Rochester, New York, USA

**Keywords:** *Citrobacter freundii*, draft genome, wastewater treatment plant, phylogenetic analysis, antimicrobial resistance genes, Stillwater, Oklahoma

## Abstract

We present draft genomes of *Citrobacter freundii* strains isolated from a model wastewater treatment plant seeded with sludge from Stillwater, Oklahoma. Analysis suggests that the Stillwater strains are related to human isolates from China and the UK. All strains also possess antimicrobial resistance genes, highlighting the potential public health implications.

## ANNOUNCEMENT

*Citrobacter freundii*, commonly found in the environment and animal gastrointestinal tracts, can cause serious infections and express multidrug resistance (1). In addition, strains harboring carbapenemase (2) and colistin resistance genes (3) represent a unique threat to treatment options for *C. freundii* infections.

We report draft genomes of five *C*. *freundii* isolated from a model wastewater treatment plant (WWTP) inoculated with sludge from the Stillwater, OK WWTP (4). All strains were isolated in 2019 on McConkey agar (37°C) and determined to be *C. freundii* utilizing matrix-assisted laser desorption/ionization-time-of-flight mass spectrometry (4). Genomic DNA was extracted from Mueller-Hinton broth cultures (OD_580nm_ = 0.7, 37°C, 200 rpm) using the GenElute Bacterial Genomic DNA Kit (Sigma-Aldrich, USA). DNA quality and concentration were assessed with NanoDrop (Thermo Fisher, MA, USA) and Qubit4 (Life Technologies, CA, USA). Illumina libraries were prepared using the Nextera XT DNA Sample Preparation Kit and indexed with the Nextera XT Index Kit (Illumina Inc., San Diego, CA, USA). Library quality and fragment size were evaluated using Qubit4 and Bioanalyzer (Agilent Technologies, CA, USA). Sequencing was performed on the Illumina MiniSeq platform.

Read quality was assessed using FastQC, and reads with scores > *Q*30 were assembled using the A5-MiSeq assembler (5). Genome annotation was performed using PROKKA (6) and RAST, and then all genomes were annotated with NCBI’s Prokaryotic Genome Annotation Pipeline during submission. The average nucleotide identities were determined with OrthoANI (7) to confirm isolate identities. Sequence types were identified via multilocus sequence typing (MLST, seven genes) (https://cge.food.dtu.dk/services/MLST/), while antibiotic resistant genes (ARGs) were predicted using ResFinder (8) and CARD (9). Plasmid sequences were identified with PlasmidFinder (10), and mobile elements were identified with MobileElementFinder (https://cge.food.dtu.dk/services/MobileElementFinder/). Roary version 3.13.0 (11) was used for pangenome analysis. Core-genome single nucleotide polymorphisms (SNPs) were called and filtered, and a phylogenetic tree was constructed with FastTree version 2.1.10 (12) in CSIPhylogeny version 1.4 (13) using accession number GCA_904859905.1_MSB1_1H (accession no LR890181.1) as reference. Tree visualization and editing were done with iTOL version 7 (14) and *C. freundii* genomes. All programs were utilized with set default parameters.

S2 harbored the fluoroquinolone resistance gene *qnrB38*, β-lactamase genes (*bla*) were found in S2, S3, S6, and S7, while S8 harbored three *bla* genes (Table 1). Plasmid sequences known to carry ARGs are present in S2, S3, S6, and S7 (15, 16), and S2, S3, and S8 contained transposons and/or insertion sequences (Table 1).

**TABLE 1:** Characteristics of *C. freundii* draft genomes isolated from a model wastewater plant

Isolate	Genome size (Mb)	Contig #	Coverage (X)	*N*_50_ (kb)	GC content (%)	Sequence type	Resistance genes	Genetic elements	GenBank/SRA accession numbers
S2	5.1	103	60.0	144.5	52	147	*qnrB38*, *bla*_CMY-65_	IncFIA, IncFII, Tn6196	JARESM000000000/SAMN50758258
S3	5.1	70	67.0	316.1	52	216	*bla*_CMY-48_	IncFIA, IncFII, Tn6196, ISCfr26	JARESL000000000/SAMN50758259
S6	5.3	119	63.0	141	52	112	*bla*_CMY-75_	IncFII, pKPC-CAV1321	JARESJ000000000/SAMN50758261
S7	5.3	112	62.0	141.6	52	112	*bla*_CMY-75_	IncFII, pKPC-CAV1321	JARESI000000000/SAMN50758262
S8	5.0	209	59.0	56.2	51.5	150	*bla*_CMY-79_/*bla*_CMY-116_/*bla*_CMY-189_	IS26	JARESH000000000/SAMN50758263

**Fig 1:**
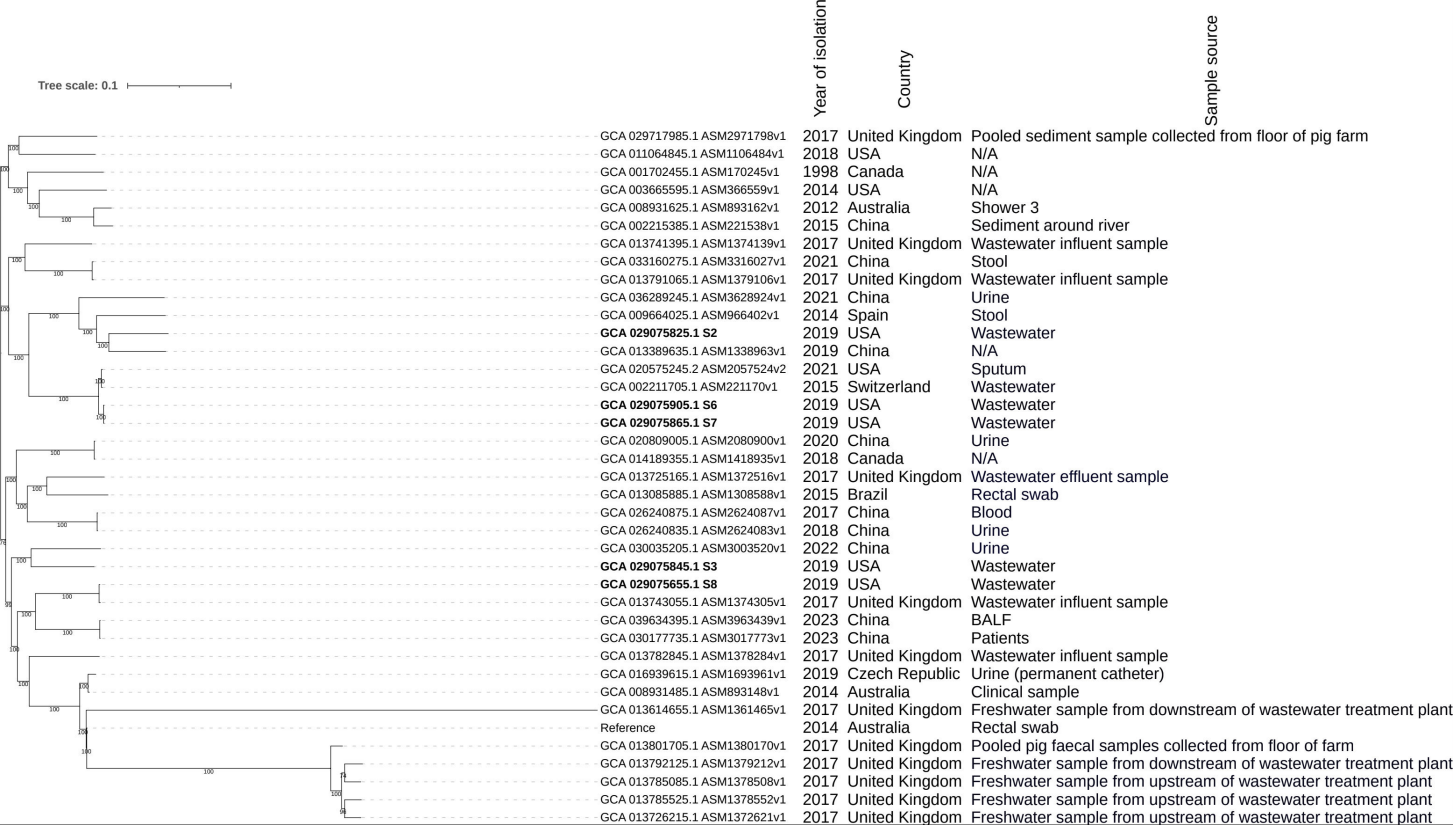
Core-genome SNP-based maximum likelihood phylogenetic tree of 39 *C*. *freundii* isolates. GCA_904859905.1_MSB1_1H (accession no LR890181.1) was used as a reference genome to which all other genomes were mapped for SNP identification. Branch lengths indicate genetic distance. Bootstrap values are shown on the nodes to represent support for branch patterns. Genomes of isolates from this study are highlighted in bold.

MLST analysis revealed that strain S2 is ST147, S3 is ST216, and S8 is ST150 (Table 1). ST150 is associated with sepsis (17) and is frequently found in Australian wastewater (18). Phylogenetic analysis grouped the Stillwater strains into two clusters (Fig. 1). S6 and S7 are both ST112 and cluster together in Fig. 1, suggesting they are clonal. S2 clustered with a Chinese human isolate (GCA 013389635.1), S3 with a Chinese urinary isolate (GCA 030035205.1), and S8 with a UK wastewater strain (GCA 013743055.1) (Fig. 1).

Our findings concur with previous work demonstrating multidrug-resistant *Citrobacter* spp. in WWTPs (19, 20) and support the importance of WWTP monitoring to mitigate resistance spread and protect public health.

## Data Availability

Draft genome sequences and raw sequencing data are available under BioProject PRJNA937283 in the NCBI databases, and the accession numbers are listed in Table 1.
